# Paired EMI-HIMU hotspots in the South Atlantic—Starting plume heads trigger compositionally distinct secondary plumes?

**DOI:** 10.1126/sciadv.aba0282

**Published:** 2020-07-08

**Authors:** S. Homrighausen, K. Hoernle, H. Zhou, J. Geldmacher, J-A. Wartho, F. Hauff, R. Werner, S. Jung, J. P. Morgan

**Affiliations:** 1 GEOMAR Helmholtz-Zentrum für Ozeanforschung Kiel, Wischhofstr. 1-3, 24148 Kiel, Germany.; 2 Institut für Geowissenschaften, Christian-Albrechts Universität zu Kiel, Ludewig-Meyn-Str. 10, 24118 Kiel, Germany.; 3 Mineralogisch-Petrographisches Institut, Universität Hamburg, 20146 Hamburg, Germany.; 4 Department of Ocean Science and Engineering SUSTech Shenzhen, China.

## Abstract

Age-progressive volcanism is generally accepted as the surface expression of deep-rooted mantle plumes, which are enigmatically linked with the African and Pacific large low–shear velocity provinces (LLSVPs). We present geochemical and geochronological data collected from the oldest portions of the age-progressive enriched mantle one (EMI)-type Tristan-Gough track. They are part of a 30- to 40-million year younger age-progressive hotspot track with St. Helena HIMU (high time-integrated ^238^U/^204^Pb) composition, which is also observed at the EMI-type Shona hotspot track in the southernmost Atlantic. Whereas the primary EMI-type hotspots overlie the margin of the African LLSVP, the HIMU-type hotspots are located above a central portion of the African LLSVP, reflecting a large-scale geochemical zonation. We propose that extraction of large volumes of EMI-type mantle from the margin of the LLSVP by primary plume heads triggered upwelling of HIMU material from a more internal domain of the LLSVP, forming secondary plumes.

## Teaser

Initiation of Tristan-Gough and Shona EMI plumes triggers secondary HIMU plumes, generating overlapping EMI-HIMU hotspot tracks.

## INTRODUCTION

The geochemical variability of oceanic lavas primarily reflects the heterogeneity of the Earth’s mantle, which requires at least four end members to explain the observed compositional range in Sr-Nd-Pb-Hf isotope ratios, known as depleted mantle (DM), enriched mantle one and two (EMI and EMII), and high μ = ^238^U/^204^Pb [HIMU; (*1*)]. The distinct geochemical signatures are generally attributed to a combination of continuous upper mantle depletion through melt extraction to form DM, whereas the origin of EMI is widely associated with recycling of continental lithospheric material [lower continental crust and/or enriched subcontinental lithospheric mantle (SCLM) (*1*)] and the origin of HIMU through the long-term [≥2 billion years (Ga)] recycling of oceanic crust or metasomatized SCLM through the lower mantle (*1*–*3*).

Global tomography images broad conduit-like, low-velocity anomalies ascending from the base of the lower mantle to the base of the lithosphere beneath ocean islands, such as St. Helena, whereas other lower mantle low-velocity anomalies are dome-like and appear to ascend only to depths of ~1000 km, such as beneath the Tristan-Gough hotspot (*4*). Above these dome-like lower-mantle structures, smaller conduit-like structures can be distinguished at shallower depths, such as beneath Tristan da Cunha to depths of ~500 km (*5*). These low-velocity seismic anomalies are believed to reflect active upwellings derived from the base of the lower mantle. Geochemical data confirm that several deep-rooted mantle plumes carry signals from recycled materials (i.e., EM and HIMU) and/or from a presumably primordial reservoir from the lower mantle, indicated by high ^3^He/^4^He and low ^182^W/^184^W ratios in the erupted plume-derived lavas (*6*, *7*). The recorded mantle heterogeneities occur at a variety of length scales, but the distribution of the geochemically distinct domains in the mantle is poorly constrained.

The majority of mantle plumes [e.g., 38 of the investigated 42 oceanic hotspots; Jackson *et al*. (*6*)] can be correlated with the two continent-sized Pacific and African large low–shear velocity provinces (LLSVPs) at the base of the lower mantle (*6*, *8*, *9*). The origin and nature of the LLSVPs are controversial, but seismic data and numerical modeling indicate that these structures are warmer and/or compositionally distinct to the surrounding mantle and most likely are partly composed of primordial and recycled material [e.g., (*9*–*11*)], therefore representing a possible source for the observed mantle plume heterogeneities [e.g., (*6*, *12*–*15*)].

**Fig. 1:**
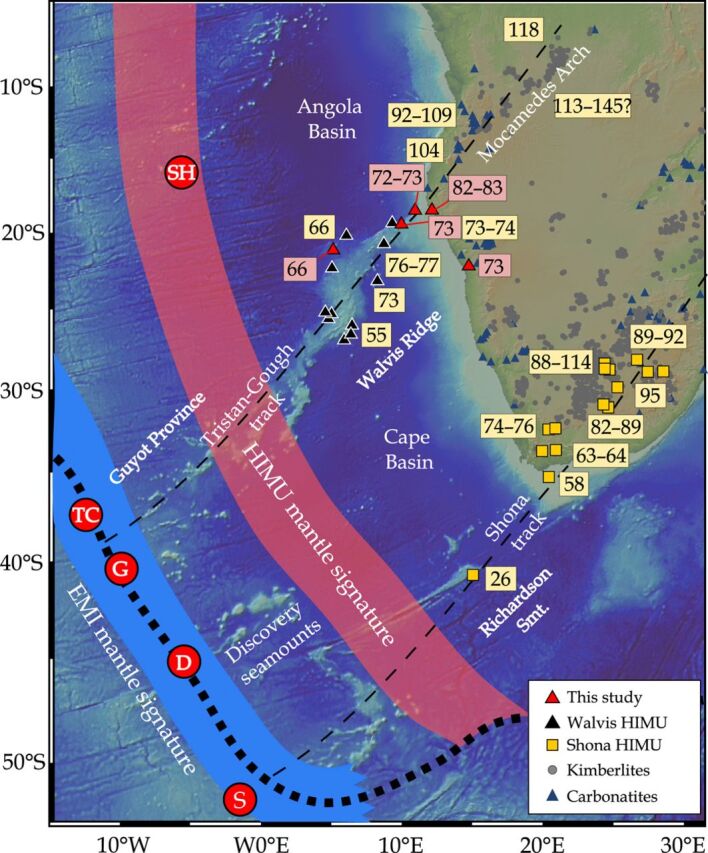
Bathymetric map of the South Atlantic. The map shows current hotspot locations (red circles: SH, St. Helena; TC, Tristan da Cunha; G, Gough; D, Discovery; and S, Shona), the Tristan-Gough and Shona hotspot tracks (thin dashed lines), the HIMU-type Walvis-Mocamedes (triangles), and Richardson–South Africa hotspot volcanism (squares). The thick dotted line defines the 1% ∂*V*s contour of the outer margin of the African LLSVP. HIMU ages are shown with pink backgrounds (this study) and yellow backgrounds [literature data (*21*, *23*, *24*, *26*–*29*, *34*, *46*, *50*–*55*)]. The blue and pink bands define the source regions of EMI- and HIMU-type plumes, respectively. Location of kimberlites and carbonatites (*56*, *57*). Figure made with GeoMapApp (www.geomapapp.org).

In the South Atlantic, the Tristan-Gough, Discovery, and Shona hotspots are characterized by a common (Gough-type) EMI geochemical component (*16*). The combination of geochronological, geochemical, numerical, and seismic studies provides evidence that these age-progressive hotspots are derived from the lower mantle [(*4*, *5*, *17*); see detailed discussion in (*16*)]. The submarine Tristan-Gough hotspot track, comprising the Walvis Ridge and Rio Grande Rise (separated by seafloor spreading) and the Guyot Province (extending from and to the southwest of the Walvis Ridge), connects the Parana/Etendeka flood basalt provinces with the volcanically active Tristan da Cunha and Gough ocean island groups (Fig. 1). At the southwest end of the Walvis Ridge, the hotspot track becomes geochemically zoned and then bifurcates into two subtracks toward (i) Gough island with a Gough-type EMI composition, which represents the long-lived compositional type that also dominates the older hotspot volcanism, and (ii) the Tristan da Cunha island group with an overall more-depleted isotopic composition, except in ^208^Pb/^204^Pb (at a given ^206^Pb/^204^Pb ratio) (*13*, *14*, *16*). This ~70–million year (Ma) spatial geochemical zonation of the hotspot track has been explained by an azimuthally asymmetric zoning of the plume conduit that samples and preserves mantle heterogeneities at the base of the lower mantle (*13*, *14*, *18*). Because the EMI-type hotspots overlie the margin of the African LLSVP (Fig. 1), it has been proposed that the Gough-type component samples the LLSVP, while the Tristan-type component is derived from the adjacent ambient mantle (*13*, *14*). Whether mantle plumes simultaneously sample compositionally distinct materials from both within and outside of the LLSVP and preserve this spatial geochemical heterogeneity during ascent is still controversial (*9*, *12*, *15*, *16*, *19*, *20*).

**Fig. 2:**
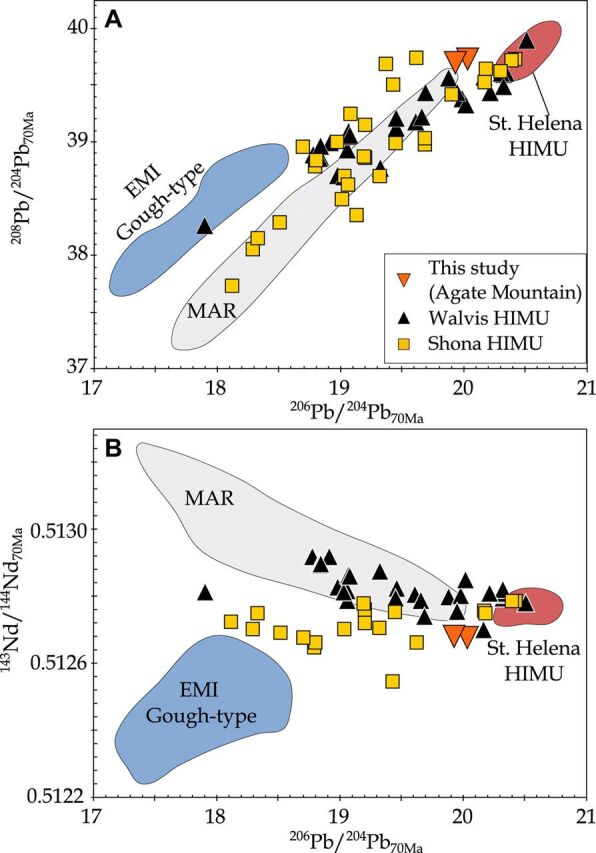
Radiogenic isotope ratio diagrams of South Atlantic and southwest African intraplate lavas. Initial isotope ratios projected to 70 Ma using proposed source parent/daughter ratios (see Materials and Methods) for HIMU lavas on ^206^Pb/^204^Pb_70Ma_ versus (**A**) ^208^Pb/^204^Pb_70Ma_ and (**B**) ^143^Nd/^144^Nd_70Ma_ diagrams. Walvis HIMU includes data from the Walvis Ridge [this study and (*3*, *21*)] and its onshore projection into northwest Namibia (Agate Mountain, this study). Shona HIMU is from the Richardson Guyot (*3*) and the onshore projection of the Shona Gough-type EMI hotspot track into South Africa (*23*, *24*, *35*–*37*). Note that the majority of lavas cannot be defined senso stricto as a St. Helena–type HIMU end member composition, but the geochemical array can be explained by mixing of a St. Helena–type HIMU end member composition with an enriched Mid Ocean Ridge Basalt component or through a mixture of depleted MORB-source mantle with Gough-type EMI plume material (*21*). Fields for the Mid-Atlantic Ridge [MAR (*58*)], Gough-type EMI [including lavas from the Tristan-Gough, Discovery, and Shona tracks; (*16*) and references therein], and St. Helena Island (*59*, *60*) are shown for comparison.

**Fig. 3:**
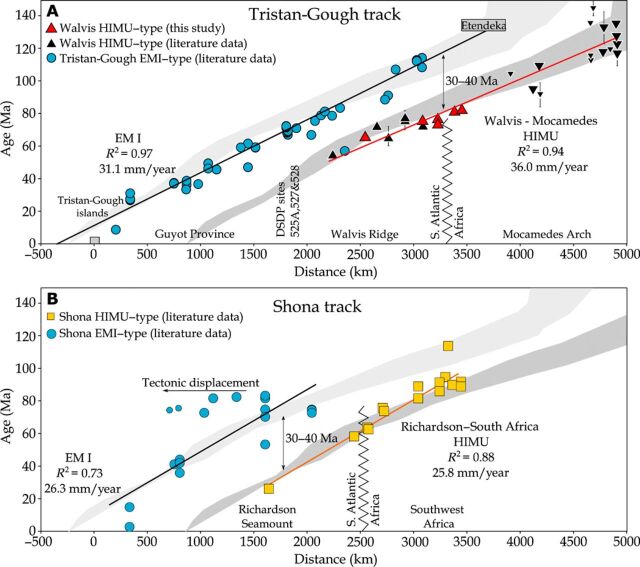
Temporal evolution of the EMI- and HIMU-like lavas. Distance versus ages for samples from (**A**) the Tristan-Gough [EMI-type; (*16*) and references therein] and Walvis-Mocamedes [HIMU-type, this study and (*21*, *26*–*29*, *46*, *52*–*55*)] and (**B**) the Shona [EMI-type; (*22*, *55*)] and Richardson–South Africa [HIMU-type; (*3*, *23*, *24*, *34*, *50*, *51*, *61*, *62*)] hotspot tracks. The sample locations were projected orthogonally to the proposed hotspot tracks (Fig. 1). The solid black and red lines display the average calculated age progressions of the EMI-type lavas and younger HIMU-type lavas, respectively. Note that the Walvis-Mocamedes HIMU age progression is calculated using the HIMU-type lavas from the Walvis Ridge, Namibia, and Mocamedes Arch (Angola; large black inverted triangles). The small black inverted triangles display Mocamedes Arch ages that have no geochemical data, which were not used for the plate velocity calculation, but fit the age progression. Errors for the ages are within the symbol size unless denoted by a vertical bar. The Shona EMI-type track was affected by movement along the Agulhas-Falkland fracture zone and spreading along the Agulhas Spreading Center (*22*). The two samples affected most by the tectonic displacement (small blue circles) were excluded for the calculated age progression. The gray arrays display the range of plate velocities derived from plate motion models for the respective hotspots (*47*–*49*).

Recently, it was found that stratigraphically younger (post-erosional) seamounts than the EMI-type Walvis Ridge basement have a distinct HIMU-type composition (Fig. 2) (*21*). ^40^Ar/^39^Ar ages show that these HIMU-type seamounts are 30 to 40 Ma younger than the EMI basement at any given location (Fig. 3) (*21*). The isotope data for these post-erosional HIMU-type seamounts form a distinct compositional array compared to the EMI-type basement (Fig. 2), most likely reflecting a two-component mixing of a St. Helena–type HIMU end member composition with an enriched mid-ocean ridge basalt–type composition (*21*).

On the Richardson Guyot, belonging to the nearby Shona hotspot track, small cones have been sampled with St. Helena–type HIMU composition and an age of 30 to 50 Ma younger than the underlying Gough-type EMI lavas (*3*). Neither of the older two underlying EMI hotspot tracks (Tristan-Gough or Shona) show any isotopic evidence of HIMU-type material being involved during their formation (*14*, *16*, *22*). As a result of the temporal difference and plate motion, the HIMU source reservoir must be spatially separated from the EMI source (*21*). In addition, alkaline lavas, carbonatites, and kimberlites with HIMU-type isotopic compositions occur in southwest Africa (Fig. 1) [e.g., (*21*, *23*–*26*)]. Thus far, the relationship between the EMI and HIMU lavas in the South Atlantic and southwest Africa has remained enigmatic.

In this study, we report geochemical and ^40^Ar/^39^Ar age data from the carbonatite/phonolite Agate Mountain complex near the northwest Namibian coast, and additional ^40^Ar/^39^Ar ages from St. Helena–type HIMU late-stage seamounts on the Walvis Ridge obtained during *R/V SONNE SO233* cruise. Our data, combined with literature data [e.g., (*21*, *23*, *26*–*29*)], show that the HIMU volcanism is age-progressive and allows us to constrain the spatial structure of compositionally different source reservoirs in the lower mantle and the origin of the overlapping HIMU hotspot tracks.

## RESULTS

The geochronological and geochemical data files are provided in the Supplementary Materials (sections S1 to S3). The carbonatitic Agate Mountain complex lies on the continental extension of the Tristan-Gough hotspot track and is intruded by late-stage phonolitic dikes and plugs [SiO_2_ = 55.6 to 56.8 weight % (wt %), total alkalis = 15.5 to 16.2 wt %; section S1]. The radiogenic isotope ratios of the phonolites are distinct from the widespread, surrounding EMI-type Etendeka flood basalt (Tristan-Gough hotspot) volcanism, but are similar to the composition of the St. Helena Island and the Walvis HIMU seamounts (Fig. 2).

The groundmass ^40^Ar/^39^Ar weighted mean ages (WMAs) of the four new Walvis Ridge seamount samples range from 65.50 ± 0.77 Ma to 73.4 ± 1.2 Ma (all uncertainties are 2σ unless otherwise stated) and lie within the age range of previously published Walvis HIMU volcanism (Fig. 3 and fig. S2). Two phonolite samples from Agate Mountain yield K-feldspar ^40^Ar/^39^Ar plateau ages ranging from 81.67 ± 0.36 and 82.96 ± 0.30 Ma (section S2). The phonolitic dikes are substantially younger than the surrounding EMI-type Etendeka flood basalt volcanism [~132 Ma (*30*)] but are slightly older than the oldest Walvis HIMU lavas (<73 Ma; Fig. 3).

## DISCUSSION AND CONCLUSION

Our geochemical and geochronological data from the Agate Mountain complex are consistent with it forming a continental extension of the submarine HIMU volcanism of the Walvis Ridge onto the Namibian coast. The Mocamedes Arch in central Angola also lies on a northeast extension of the Walvis Ridge, i.e., in the direction of plate motion (Fig. 1). Several magmatic complexes in the southwest portion of the Mocamedes Arch underwent two magmatic stages (*28*). The oldest magmatic activity at ~130 Ma is related to the Etendeka flood basalt event (*28*), whereas the younger magmatic stage at ~95 Ma could be associated with the HIMU volcanism. The Sr-Nd isotope ratios of these lavas, as well as some Cretaceous carbonatites (*25*) and kimberlites (*26*) in the northeast portion of the Mocamedes Arch lie within the compositional array of the Walvis HIMU-type lavas [^87^Sr/^86^Sr_70Ma_ = 0.7029 to 0.7045 and ^143^Nd/^144^Nd_70Ma_ = 0.5126 to 0.5128; (*25*, *26*)]. Combining all available geochronological data for samples with a HIMU affinity, the combined “Walvis-Mocamedes” HIMU lineament forms an age-progressive trend with an average plate velocity of 36.0 mm/year (*n* = 17, *R*^2^ = 0.94), similar to the submarine EMI-type age progression of the Tristan-Gough hotspot track over the last 115 Ma (31.1 mm/year, *n* = 48, *R*^2^ = 0.97; Fig. 3A).

Previously, it was proposed that HIMU volcanism in southwest Africa and the South Atlantic could be derived from lithospheric sources, melting of diffuse, scattered blob-like upwellings, or the flow of plume material from a hotspot beneath Africa in a channel beneath the Tristan-Gough hotspot track (*21*, *23*). Recent studies suggest that the African SCLM was metasomatized by HIMU-type asthenospheric melts, shortly before the eruption of HIMU-type lavas in southwest Africa (*31*, *32*). Scattered blob-like upwellings or melt migration along the base of the lithosphere or along weak lithospheric zones, as well as tectonic complexities, could explain the larger spatial and temporal scatter of volcanism along the continental parts of the HIMU lineaments (Fig. 3). These models, however, cannot explain (i) the systematic age progression in the direction of and at a similar rate to the plate motion and (ii) that the hotspot track crosses the continent-ocean boundary. A relatively stationary melting anomaly in the asthenosphere (plume), however, can explain these observations (Fig. 3A).

The HIMU reservoir is believed to be located in the lower mantle [(*2*, *3*) and references therein]. A potential HIMU-like reservoir in the transition zone, on the other hand, has been recently proposed by Mazza *et al*. (*33*). Lavas interpreted to be derived from the transition zone, however, are characterized by unusually high ^206^Pb/^204^Pb ratios at low ^207^Pb/^204^Pb ratios. Thus, the Bermuda-type HIMU end member is distinct from the St. Helena–type HIMU end member composition. To account for the specific geochemical signature of the St. Helena–type HIMU end member, a reservoir formation age and isolation of 1 to >2.7 Ga are required [(*2*, *3*) and references therein]. The transition zone, on the other hand, is continuously disturbed by subducting slabs and upwelling mantle plumes, and it seems questionable if relatively large portions remain sufficiently isolated for more than 1 Ga. Furthermore, seismic tomography has imaged a continuous conduit-like, low-velocity anomaly extending from the base of the lithosphere beneath St. Helena through the transition zone to the base of the lower mantle [e.g., (*4*)], providing strong evidence that the St. Helena HIMU reservoir is located in the lower mantle. Therefore, the overlapping but compositionally distinct (EMI and HIMU) age-progressive volcanic tracks are most likely formed by two distinct, deep-rooted mantle plumes.

Along the Shona hotspot track, lavas from a late-stage cone on the Richardson Guyot (*3*), the Alphard Bank and Western Cape Province (*23*), and several group I kimberlites in southwest Africa [i.e., Monastery, Kaalvallei, Frank Smith, Thaba Putsoa, De Beers, Jagersfontain, and Wesselton; (*24*, *34*–*37*)] all share a common St. Helena–type HIMU end member component (Fig. 2) (*21*). Similar to the Walvis-Mocamedes HIMU lineament, these HIMU-like lavas form a linear array on multiple isotope diagrams, which is distinct from the older EMI-type lavas (Fig. 2). The array most likely reflects mixing of St. Helena–type HIMU with an enriched Mid-Ocean Ridge Basalt component, which could reflect contamination of depleted upper mantle in the asthenosphere or lithosphere by the earlier EMI-type plume (Fig. 2) (*21*). The HIMU-type lavas overlie and continue to the northeast of the older part of the Gough-type EMI Shona hotspot track (Fig. 1). This “Richardson–South Africa” HIMU lineament forms an age-progressive trend (~25.8 mm/year; *n* = 16, *R*^2^ = 0.88), which is comparable to the age progression derived from the underlying Shona EMI-type lavas (26.3 mm/year, *n* = 16, *R*^2^ = 0.73; Fig. 3). The overall slower age progressions of the Shona and Richardson–South Africa tracks (compared to the Walvis EMI basement and HIMU Walvis-Mocamedes tracks) reflect the large uncertainties for these overall shorter, poorly sampled, and tectonically disturbed hotspot tracks (*22*). Thus, the EMI Shona and HIMU Richardson–South Africa lineaments represent the second overlapping (but geochemically distinct) hotspot tracks in the South Atlantic, suggesting that the pairing of Gough-type EMI primary and St. Helena–type HIMU secondary hotspot tracks is not fortuitous and reflects a similar spatial-structural relationship in the plume source regions in the lower mantle.

All three primary EMI-type mantle plumes in the South Atlantic (Tristan-Gough, Discovery, and Shona) lie above the steep outer margin of the African LLSVP (Fig. 1). To form paired, overlapping hotspot tracks, the EMI and HIMU mantle upwellings and their sources need to be offset in the direction of the African Plate motion, which is nearly perpendicular to the southwest African LLSVP margin (Fig. 1). The systematic temporal offset of ~30 to 40 Ma between the primary EMI and secondary HIMU volcanism at a given location along a volcanic track (Fig. 3) corresponds to a spatial offset of the HIMU from the EMI melting anomaly (and source) of ~900 to 1200 km to the northeast in the direction of plate motion (Fig. 1; using an average plate velocity of 30 mm/year). The St. Helena hotspot shows a similar displacement from the margin of the LLSVP (*6*). The spatial arrangement between the paired EMI-HIMU hotspots must reflect large-scale and long-lasting geochemical zonation subparallel to the African LLSVP margin in the hotspot sources (Fig. 1).

Because the Gough-type EMI plumes are located above the margin of the African LLSVP, their source reservoir could either be located within the LLSVP or represent the ambient mantle flowing against its steep outer margin (*13*, *14*, *16*). The common St. Helena–type HIMU reservoir, however, must be located within the interior or on top of the LLSVP (*21*), because such material must be isolated from the convecting mantle for periods of 1 to >2.5 Ga [(*3*) and references therein]. The idea that LLSVP material is entrained by mantle plumes is debated [e.g., (*8*, *9*, *11*, *12*, *15*, *16*, *19*, *20*)], but geochemically zoned hotspot tracks (such as Hawaii and Tristan/Gough) are commonly explained by sampling of enriched material from the LLSVP margin (e.g., Gough-type material) and of more depleted material from the ambient mantle or subducted material from outside of the LLSVP [e.g., Tristan-type; (*12*–*15*)] with preservation of the source heterogeneity (sampling of distinct geochemical domains) by laminar flow within the plume conduit (*18*). High ^3^He/^4^He and low ^182^W/^184^W ratios in some Gough-type EMI lavas [e.g., (*7*, *16*, *17*)] confirm primordial material entrainment in the South Atlantic EMI-type mantle plumes.

**Fig. 4:**
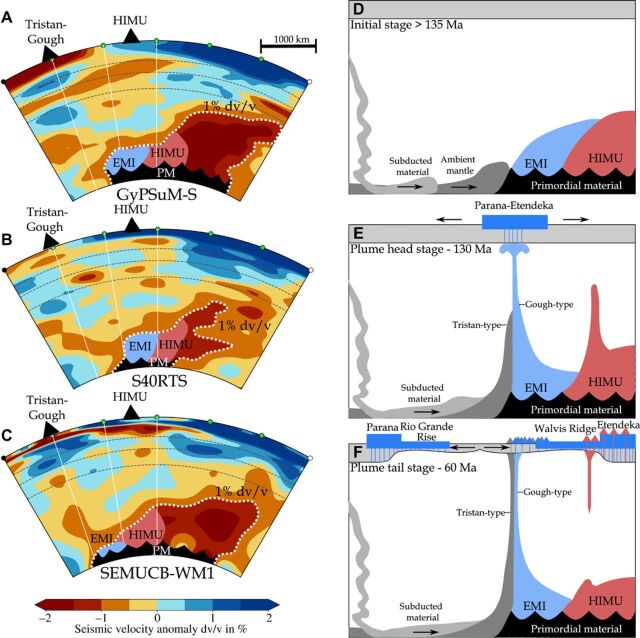
Tomographic profiles through the mantle beneath the southern Atlantic and Africa and our preferred model for the formation of compositionally distinct paired hotspot tracks. (**A** to **C**) Three tomographic models [GyPYSuM-S (*63*), S40RTS (*64*), and SEMUCB-WM1 (*4*)], displaying the present-day seismic structure beneath the South Atlantic and South Africa from latitude 50°S, longitude 20°E to latitude 10°S, longitude 35°W. Note that all models show a similar LLSVP shape (the 1% ∂Vs (dv/v) contour is shown by the white dotted line) and lateral variations, with a step in the LLSVP surface at ~1000 to 1500 km from the western margin of the LLSVP, which could represent the remnants of a secondary plume generation zone. (**D** to **F**) A possible schematic model to explain the large-scale geochemical zonation in the South Atlantic and the origin of paired age-progressive hotspot tracks. The compositionally layered LLSVP model [after Ballmer *et al*. (*10*)] is divided into a bottom layer, consisting of primordial material (PM), that stabilizes the LLSVP, and a shallow domain of accumulated recycled material. The schematic model is divided into three stages: (D) The “>135 Ma initial stage” showing a possible African LLSVP structure before the Tristan-Gough mantle plume rose. We have divided the upper LLSVP layer into marginal EMI-type and more central HIMU-type subdomains, which reflect the observed geographic separation of similarly aged HIMU and EMI intraplate lavas. (E) With the initiation of the Tristan-Gough mantle plume at ~130 Ma, large amounts of EMI-type mantle from the marginal EMI domain ascended in the starting plume head and early plume tail, exposing the HIMU domain in the interior of the LLSVP. The secondary HIMU-type mantle plume initiates after 130 Ma and continues until (F) ~55 Ma when the topology of the shallow LLSVP domain is smoothed out and the rather short-lived secondary HIMU mantle plume dies, whereas the primary EMI mantle plume at the steep LLSVP margin remains active, but no longer removes much material from the LLSVP. The exact EMI-HIMU distribution in diagrams (D to F) is speculative, but the large-scale geochemical zonation of South Atlantic intraplate lavas points to distinct EMI- and HIMU-type domains at the southwest margin of the African LLSVP. The Tristan-Gough plume becomes zoned at ~70 Ma, incorporating Gough-type EMI from the LLSVP (light blue) and Tristan-type ambient mantle [dark gray (*13*, *14*)]. The tomographic images were prepared using the web-based tool from Hosseini *et al*. (*65*).

Seismic heterogeneities within the LLSVPs most likely reflect compositional heterogeneities [e.g., (*9*, *10*, *20*)]. Numerical simulations indicate that subducted material could be added to possibly primordial LLSVPs over time (*11*, *20*), but this interaction is unclear [e.g., (*9*–*11*)]. The compositionally layered LLSVP model of Ballmer *et al*. (*10*), for example, suggests a primordial bottom layer and an overlying upper domain consisting of accumulated recycled materials (Fig. 4; i.e., EM and HIMU). The numerical simulations from Li *et al*. (*11*), on the other hand, suggest that subducted slabs continuously enter the primordial LLSVPs, leading to multiscale heterogeneities. Numerical simulations [e.g., (*9*, *20*)] also predict entrainment of compositionally heterogeneous LLSVP material into rising mantle plumes. In accordance with these simulations, upwellings from the more internal portions of the LLSVP (900 to 1200 km away from the margin) would likely tap a distinct geochemical reservoir compared to upwellings from the margin. Therefore, a compositionally layered LLSVP (*10*) or a heterogeneous mixture of recycled and primordial materials (*11*) could explain the large-scale geochemical variation observed in intraplate volcanism in the South Atlantic and southwest Africa.

An important question is why and how the secondary HIMU plumes were initiated. Accepting that EMI and HIMU are stored within and/or on the LLSVP, we present a simple model that explains the temporal geochemical evolution of intraplate volcanism in the South Atlantic and southwest Africa, in particular the spatially overlapping but compositionally distinct hotspot tracks (Fig. 4). This model is also applicable if the EMI and HIMU materials are located in distinct reservoirs in the ambient mantle around the LLSVP, with EMI material deflected upward at the outermost boundary at the base of the LLSVP and HIMU material being deflected upward at an internal step in the LLSVP. We, however, prefer isolation of these end members within or on the LLSVP to allow sufficient radiogenic ingrowth to generate their end member isotopic compositions.

Paleogeographic reconstructions (*8*) and geochemical evidence (*14*, *38*) indicate that the Etendeka/Parana flood basalt provinces represent the initial stage of the Tristan-Gough plume (*39*) and suggest that the Karoo flood basalt province represents the initial stage of the Shona plume (*8*). Therefore, both primary plumes (forming the EMI-type Shona and Tristan-Gough hotpot tracks) appear to have initiated with a flood basalt event. Because the oldest reported HIMU volcanism from both subsequently formed overlapping HIMU volcanic tracks is younger than the EMI-type volcanism (Fig. 3), we propose that the sudden removal of large amounts of material from an outer EMI reservoir by the plume heads could have generated internal instabilities that triggered smaller, transient secondary plumes (without large initial heads) upslope of the LLSVP margin (Fig. 4). Numerical simulations show that instabilities can occur at the top of the LLSVP depending on its morphology (*9*, *10*). There is a steep step ~1000 to 1500 km northeast of the outer margin of the African LLSVP, which coincides with the offset distance between the EMI and HIMU hotspot volcanism (Figs. 3 and 4). This step (change in LLSVP morphology) could represent the generation zone of the secondary HIMU upwelling. We speculate that rapid removal of EMI-type mantle from the edge of the LLSVP during the plume head stage could have formed the step ~1000 to 1500 km from the LLSVP outer margin, exposing hot HIMU material in the interior or upper layer of the LLSVP to the cooler ambient mantle material surrounding the LLSVP. The temperature contrast would have increased the effective buoyancy of the HIMU material, leading to the rise of small positively buoyant secondary HIMU plumes. Alternatively, steep steps in internal sections of the LLSVP could act as “plume generation zones” similar to the sharp boundaries at the outer margins of the LLSVPs, where flow along the base of the lower mantle could induce mantle upwellings when they are diverted upward at the LLSVP margins (*19*).

A narrow linear track formed by several stationary melt anomalies aligned in the direction of plate motion has been previously proposed for the Cook-Austral Archipelago. The geochemically distinct Samoan (EMI-EMII and HIMU-like), Rarotonga (EMI), Macdonald (pimarily HIMU-like), and Arago-Rurutu (HIMU-like) volcanic chains [arranged with increasing distance from the edge to the center of the LLSVP; SMEAN2 model distance from (*6*)] appear to define distinct age-progressive hotspot tracks that were superimposed on each other and aligned in the direction of plate motion since ~50 to 47 Ma (*40*). End member HIMU- and EM-type compositions are also present in these regions. The more gradational geochemical variations may, in part, reflect that the “hotspot profile” is oblique to the Pacific LLSVP boundary, rather than perpendicular as is the case beneath the South Atlantic. Nevertheless, it appears that EM-type material in Pacific zoned hotspots occurs preferentially above the margins of the Pacific LLSVP, for example, as seen at the Hawaiian and Marquesas hotspots [the EMI-type Loa and Motu subtracks, respectively, are located on the LLSVP side and the more depleted Kea and Nuku toward the ambient mantle; (*12*, *15*)].

The large-scale geochemical zonation in southern Atlantic and African intraplate volcanism oriented parallel to the LLSVP margin provides evidence for a heterogeneous African LLSVP with domains of recycled material. Because the South Atlantic EMI-type hotspot tracks show no evidence of St. Helena–type HIMU material, the material transfer and mixing between the distinct reservoirs in/on the African LLSVP appear to be extremely limited. The removal of material from a marginal reservoir of the LLSVP during a flood basalt event and subsequent hotspot track volcanism could cause internal instabilities in/on the LLSVP resulting in the formation of a second plume generation zone that could tap another geochemically distinct large-scale lower-mantle reservoir. The LLSVP, therefore, seems to be a dynamic structure with changing morphology over time. Geochemically zoned hotspot tracks could also potentially form at steep margins within internal portions of the LLSVP [e.g., Samoa and Marquesas (*12*)].

## MATERIALS AND METHODS

### Sample preparation

Alteration rinds and encrustations were removed from the rock samples to prepare rock chips, powders, and mineral separates for analysis. After drying the rock slabs at 50°C, they were crushed in a steel jaw crusher, dry sieved, and cleaned with deionized water in an ultrasonic bath to remove dust. For the geochemical analyses, the 0.5- to 1-mm size fractions were carefully handpicked under a binocular microscope to obtain the freshest whole rock material. Several grams of these picked rock chips were milled to a fine powder using an agate mortar grinder and an agate planetary ball mill. The powder was used for major and trace element analysis, and the remaining half of the rock chips were used for Sr-Nd-Pb isotopic analysis.

For ^40^Ar/^39^Ar dating, the basalt groundmass separates (SO233 DR75-1, DR89-1, DR90-1, DR90-2, and NAM61) were separated using a Frantz magnet separator to enrich the plagioclase-rich portion of the 0.25- to 0.5-mm size fraction. The plagioclase-enriched groundmass separates were treated with 3.5 N HCl acid for 1 hour, followed by 1 N HNO_3_ acid for 1 hour, and finally washed with distilled water for 1 hour at 65°C in an ultrasonic bath. Between each step of the acid treatment and after the last ultrasonic bath treatment in water, the groundmass was rinsed five times with distilled water. Samples NM31 and NM32 were also hot acid–treated (*41*), and the K-feldspars were handpicked under a binocular microscope from the 0.25- to 0.5-mm size fraction. The K-feldspar separates were additionally treated with 5% hydrofluoric acid for 10 min, rinsed with distilled water, cleaned with distilled water for 4 min using an ultrasonic stick, and dried overnight at 50°C in an oven. The mineral separates were all finally cleaned in American Chemical Society (ACS)–grade acetone, followed by rinsing in distilled water and drying overnight at 50°C in an oven.

### Geochemical methods

Major element compositions were measured on fused beads at the Institute of Mineralogy and Petrography, University of Hamburg, by x-ray fluorescence analysis (XRF) using MagiX Pro PW 2540. Reference materials JGB-1, JB-3, JB-2, JA-3, JG3, and JG-2 were analyzed along with the samples (section S3).

Trace element concentrations were determined by inductively coupled plasma mass spectrometry (ICP-MS) at the Institute of Geosciences, Christian-Albrechts-University of Kiel, using an Agilent 7500cs ICP-MS. Powdered sample (100 mg) was dissolved by pressure digestion and analyzed following the methods of Garbe-Schönberg (*42*). International rock standards BHVO-2, BIR-1, and BCR-2 were analyzed together with the samples, and most elements agree within 5% of the GeoReM reference values, except for Li, Cr, and Zr with larger deviations and Ta and U with <0.1 parts per million (ppm) concentration (http://georem.mpch-mainz.gwdg.de/). Reproducibility of separately replicated samples is typically better than 5% for reference materials and samples (section S3).

Radiogenic isotope analyses were carried out at GEOMAR Helmholtz Centre for Ocean Research Kiel using a Thermo Fisher Scientific TRITON+ thermal ionization mass spectrometer (TIMS) for Sr-Nd-Pb analyses operating in static multicollection mode. Sr, Nd, and Pb chemistry was conducted on 100 to 250 mg of whole-rock chips. Before dissolution in a 5:1 mixture of concentrated HF and HNO_3_ acids, they were leached in 2 N HCl at 70°C for 1 hour and then triple rinsed in 18.2 megohms of deionized water. Ion chromatography methods followed established standard procedures of Hoernle *et al*. (*43*). Nd and Sr. ratios were normalized within the analytical run to values of ^146^Nd/^144^Nd = 0.7219 and ^86^Sr/^88^Sr = 0.1194, respectively. NBS987 and La Jolla reference materials were measured four to six times in each sample turret. The numerical average obtained for the standards is subtracted from the preferred standard values, and the off-set value is applied to the sample data. This measure compensates for long-term machine drift and thereby ensures maximum comparability and quality of data generated at different times. Since the installation of the instrument in 2014, sample data are reported relative to ^87^Sr/^86^Sr = 0.710250 ± 0.000009 (*n* = 536; 2 SD external reproducibility) for NBS987 and ^143^Nd/^144^Nd = 0.511850 ± 0.000007 (*n* = 347; 2 SD external reproducibility) for La Jolla. The Pb double spike (DS) technique [see Hoernle *et al*. (*44*)] was used to mass bias correct the Pb isotope ratios. DS corrected NBS981 values are ^206^Pb/^204^Pb = 16.9407 ± 0.0018, ^207^Pb/^204^Pb = 15.4976 ± 0.0018, ^208^Pb/^204^Pb = 36.7200 ± 0.0046, ^207^Pb/^206^Pb = 0.91482 ± 0.00004, and ^208^Pb/^206^Pb = 2.16756 ± 0.00008 (*n* = 116; 2 SD). The total Pb chemistry blanks were below 30 pg and are thus considered negligible. Correction for radiogenic ingrowth since eruption, followed by projection to a common age, was calculated following the method of Homrighausen *et al*. (*16*).

### ^40^Ar/^39^Ar dating methods

All the samples were irradiated with fast neutrons in the cadmium-lined in-core irradiation tube (CLICIT) reactor core position at the Oregon State University (OSU) nuclear reactor, Corvallis, USA. Samples NM31, NM32, and NAM61 were irradiated for 4 hours (Can #47), and sample DR90-1 was irradiated for 1 hour (Can #45). Taylor Creek sanidine (TCR-2) age standard (27.87 ± 0.04 Ma; 1σ error) was used to monitor the fast neutron gradient. Samples DR75-1, DR89-1 m, and DR90-2 were double-irradiated for 4 hours (Can #43) followed by 1 hour (Can #45). Double irradiation of these samples was necessary due to a series of laser and mass spectrometer breakdowns, plus reconstruction work in the GEOMAR laboratory in 2017, which resulted in >6 months elapsing since irradiation of the Can #43 Ca-bearing samples, thus allowing the majority of the ^37^Ar_Ca_ to decay away. The larger errors associated with the *J* values reflect the combined *J* values of two sets of TCR-2 age standards from Cans #43 and #45 irradiations, and the *J* values and 1σ errors for each sample are noted in section S2.

The ^40^Ar/^39^Ar analyses were all conducted at the GEOMAR Argon Geochronology in Oceanography (ARGO) Laboratory, similar to the laser step-heating technique outlined by Homrighausen *et al*. (*21*). The corrections, age determinations, and plateau and isochron plots were made using the Excel ArArCALC macro.

The ^40^Ar/^39^Ar plateau ages were determined by the following criteria: (i) a valid ^40^Ar/^39^Ar plateau age contains least three consecutive laser heating steps, comprising >50% of the ^39^Ar released, with ages overlapping within 2σ errors, and (ii) the plateau and inverse isochron ages should be concordant at the 95% confidence level. Pseudo-plateau and WMAs indicate total % ^39^Ar values of 30 to 49% and <30%, respectively. For multiple splits of the same sample (e.g., NM31), a combined ^40^Ar*/^39^Ar WMA using the plateau ages from each aliquot was calculated using Isoplot software version 4.15.

Alteration index (AI) values were determined and used as a guide to gauge the alteration/freshness of the step-heated basaltic groundmass and K-feldspar samples, using the following cutoff values: ^36^Ar/^39^Ar AI < 0.0006 for basalt and ^36^Ar/^39^Ar < 0.00006 for K-feldspar [Samrock *et al*. (*45*) and references therein]. In addition, the % radiogenic ^40^Ar (^40^Ar*) and K/Ca ratios (obtained from measured ^39^Ar_K_/^37^A_ca_ ratios) were used to check for the presence of alteration or mixed phases in the samples, respectively.

### ^40^Ar-^39^Ar dating results

The ^40^Ar-^39^Ar dating results for the individual laser step-heated samples from the Walvis Ridge (*n* = 4) and Namibia (*n* = 3) are reported in section S2.

The Walvis Ridge very altered groundmass samples yielded disturbed age spectra with older low-temperature apparent ages and younger more consistent high-temperature ages (section S2). The older low-temperature ages may reflect some incorporation of excess ^40^Ar, which cannot be confirmed due to tight clustering of the majority of the analyses on the inverse isochron plots, with either no isochrons or low (<40%) spreading factor values (section S2). Alternatively, the older low-temperature step ages may be due to the effects of ^39^Ar recoil effects from fine-grained material [e.g., (*41*)]. The quoted WMAs for these groundmass samples are all taken from the more consistent high-temperature step ages that generally correspond to higher % radiogenic ^40^Ar (^40^Ar*) values (77 to 100%) and lower ^36^Ar/^39^Ar AI ratios, which reflect degassing from fresher or less altered material (section S2). Higher K/Ca ratios for these high-temperature WMAs probably reflect degassing of plagioclase phenocrysts in these groundmass samples (section S2).

The Namibian K-feldspar samples (NM31 and NM32) yield plateau ages, and the majority of the steps show high % ^40^Ar* ratios (73 to 100%) and low ^36^Ar/^39^Ar AI ratios reflecting degassing from fresh material (section S2). Two splits of K-feldspar from sample NM31 yield plateau ages that overlap within their 2σ uncertainties; therefore, a combined weighted mean ^40^Ar*/^39^Ar age of 82.90 ± 0.24 Ma (95% confidence level) was calculated for this sample (section S2).

The Namibian basanite groundmass sample (NAM61) yielded a disturbed age spectrum from altered material. All the ^36^Ar/^39^Ar AI step values were higher than the basalt cutoff value of <0.006, and the sample also contained very high Cl values in the initial three steps, resulting in some suppression of the ^40^Ar and ^39^Ar isotope signals. However, the high-temperature steps yielded a pseudo-plateau age with higher radiogenic ^40^Ar values (71 to 97%; section S2).

### Uncertainties of plate velocity calculations

The calculated age progressions are dependent on a number of assumptions and parameters. In general, we assumed a stationary melt anomaly under a constantly moving plate over the respective time interval. Furthermore, several parameters such as (i) the proposed hotspot track, (ii) sample density, (iii) sampled magmatic stage (i.e., main or late stage), (iv) track length, and (v) age data quality significantly affect the resulting plate velocities. For example, Rohde *et al*. (*46*) used ^40^Ar-^39^Ar ages from the same sample sites but chose a slightly different hotspot track. Rohde *et al*. (*46*) calculated an age progression of 28 mm/year for the Walvis Ridge and 30 mm/year for the entire track (excluding Tristan and Gough islands). Using the most comprehensive dataset from the long-lived Tristan-Gough hotspot track, Homrighausen *et al*. (*16*) calculated an average plate velocity for the submarine samples of 31 mm/year. On the other hand, plate motion models indicate a reduction of plate velocity of ~10 mm/year during the Cenozoic (*47*–*49*). Therefore, specific time intervals of the hotspot tracks yield distinct plate velocities (Fig. 3). Furthermore, the respective hotspot tracks lie within the uncertainties of various plate motion models, which additionally confirms our interpretation of paired age-progressive hotspot tracks.

## Supplementary Material

aba0282_Section_S3.xlsx

aba0282_Section_S1.xlsx

aba0282_SM.pdf

aba0282_Section_S2.xlsx

## SUPPLEMENTARY MATERIALS

Supplementary material for this article is available at http://advances.sciencemag.org/cgi/content/full/6/28/eaba0282/DC1
