# Supplementary material for: Paired EMI-HIMU hotspots in the South Atlantic—Starting plume heads trigger compositionally distinct secondary plumes?
Source: Sci Adv. 2020 Jul 8;6(28):eaba0282. doi: 10.1126/sciadv.aba0282 (PMC7343398; doi:10.1126/sciadv.aba0282)
Supplement: aba0282_SM.pdf [file aba0282_SM.pdf]

Science Advances
AAAS

advances.sciencemag.org/cgi/content/full/6/28/eaba0282/DC1

Supplementary Materials for

Paired EMI-HIMU hotspots in the South Atlantic—Starting plume heads trigger compositionally distinct secondary plumes?

S. Homrighausen*, K. Hoernle, H. Zhou, J. Geldmacher, J-A. Wartho, F. Hauff, R. Werner, S. Jung, J. P. Morgan

*Corresponding author. Email: shomrighausen@geomar.de

Published 8 July 2020, *Sci. Adv.* **6**, eaba0282 (2020)
DOI: 10.1126/sciadv.aba0282

The PDF file includes:

Figs. S1 and S2

Other Supplementary Material for this manuscript includes the following:

(available at advances.sciencemag.org/cgi/content/full/6/28/eaba0282/DC1)

Sections S1 to S3

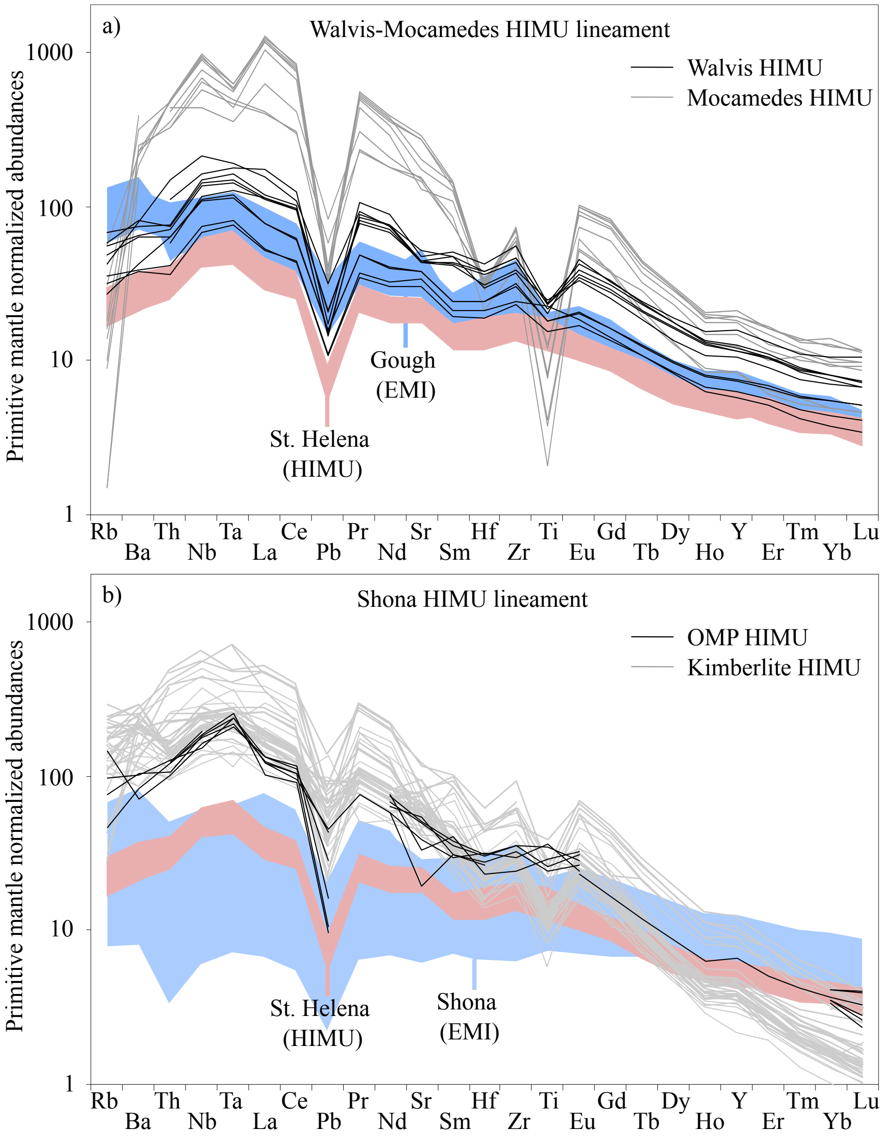

Fig. S1.

Normalized incompatible element diagram of a) the Walvis-Mocamedes HIMU lineament and b) Shona HIMU lineament compared to St. Helene HIMU composition and the respective EMI hotspot lavas. Literature data is reported in Fig. 3.

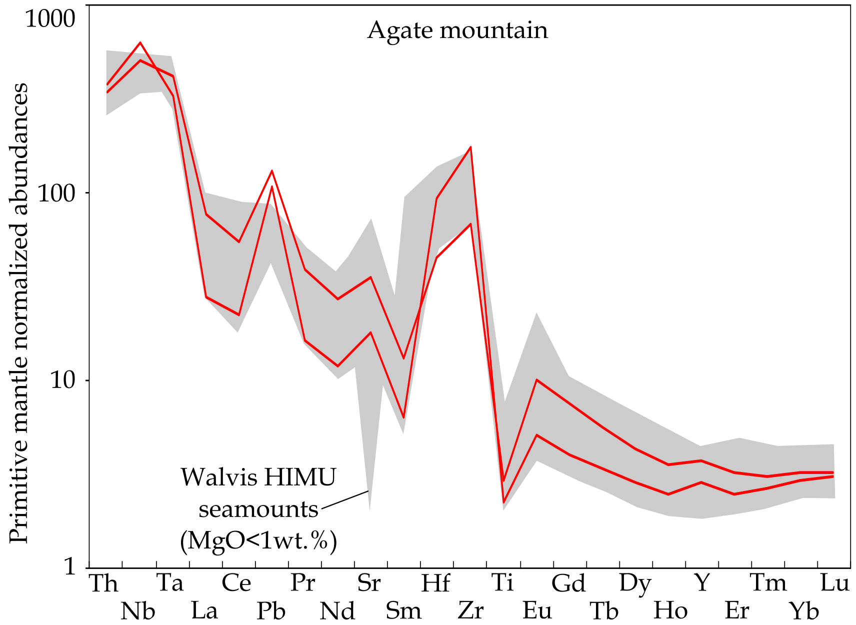

Fig. S2.

Normalized incompatible element diagram of the reported Agate mountain lavas compared to similar evolved (MgO<1wt.%) Walvis HIMU late-stage lavas. Literature data is reported in Fig. 3.
